# Elemental and isotopic analysis of leaves predicts nitrogen-fixing phenotypes

**DOI:** 10.1038/s41598-024-70412-8

**Published:** 2024-08-29

**Authors:** Joshua R. Doby, Carolina M. Siniscalchi, Mariela Pajuelo, John Krigbaum, Douglas E. Soltis, Robert P. Guralnick, Ryan A. Folk

**Affiliations:** 1 Florida Museum of Natural History, University of Florida, Gainesville, FL 32611 USA; 2 General Libraries, Mississippi State University, Mississippi State, MS 39762 USA; 3 Department of Anthropology, University of Florida, Gainesville, FL 32611 USA; 4 Department of Biology, University of Florida, Gainesville, FL 32611 USA; 5 Biodiversity Institute, University of Florida, Gainesville, FL 32611 USA; 6 Thompson Earth Systems Institute, University of Florida, Gainesville, FL 32611 USA; 7 Department of Biological Sciences, Mississippi State University, Mississippi State, MS 39762 USA

**Keywords:** Biological techniques, Ecology, Plant sciences, Biogeochemistry, Stable isotope analysis

## Abstract

Nitrogen (N)-fixing symbiosis is critical to terrestrial ecosystems, yet possession of this trait is known for few plant species. Broader presence of the symbiosis is often indirectly determined by phylogenetic relatedness to taxa investigated via manipulative experiments. This data gap may ultimately underestimate phylogenetic, spatial, and temporal variation in N-fixing symbiosis. Still needed are simpler field or collections-based approaches for inferring symbiotic status. N-fixing plants differ from non-N-fixing plants in elemental and isotopic composition, but previous investigations have not tested predictive accuracy using such proxies. Here we develop a regional field study and demonstrate a simple classification model for fixer status using nitrogen and carbon content measurements, and stable isotope ratios (δ^15^N and δ^13^C), from field-collected leaves. We used mixed models and classification approaches to demonstrate that N-fixing phenotypes can be used to predict symbiotic status; the best model required all predictors and was 80–94% accurate. Predictions were robust to environmental context variation, but we identified significant variation due to native vs. non-native (exotic) status and phylogenetic affinity. Surprisingly, N content—not δ^15^N—was the strongest predictor, suggesting that future efforts combine elemental and isotopic information. These results are valuable for understudied taxa and ecosystems, potentially allowing higher-throughput field-based N-fixer assessments.

## Introduction

Nitrogen (N) is critical for plant growth and development, but plants cannot directly use atmospheric nitrogen (N_2_) despite its abundance and must rely on bioavailable nitrogen in soil. Only plants in a few clades have evolved adaptations to take advantage of abundant atmospheric nitrogen, all of which have converged on leveraging symbioses with bacteria that fix N_2_. The most diverse and widespread group of plants to utilize this relationship is a group of angiosperms, the nitrogen fixing clade^1,2^. Approximately 52% of the members of this clade house aerobic nitrogen fixing bacteria (either rhizobia or *Frankia*), contained in root nodules, that conduct the anaerobic nitrogen fixation reaction under O_2_-protected microaerophilic conditions^3–6^. The agriculturally important legumes (Fabaceae) are the best-known members of the nitrogen fixing clade, but root nodule mediated symbiosis also occurs in nine non-legume families of plants^1^ .

Nitrogen occurs naturally as two stable isotopes: most (99.6%) naturally occurring nitrogen is ^14^N, and the remainder is ^15^N. The ability to directly access atmospheric N_2_ via symbiotic diazotrophs means that N-fixing plants not only produce proportionally more foliar nitrogen, but also should have differing δ^15^N values (expressed as ^15^N/^14^N of a sample vs. an international standard–AIR, or atmospheric N_2_) compared to non-N-fixing plants. This occurs because the abundance of ^15^N generally differs between the atmosphere and soils^7^, and therefore the ratio of these isotopes (^15^N/^14^N) indicates the source of nitrogen as the result of recent atmospheric fixation or cycling of bioavailable soil nitrogen.

Because nitrogen-fixing symbiosis has implications for both total N and ^15^N, both elemental and isotopic information could be used to infer whether the plant acquired bioavailable N from the soil or atmosphere^8,9^. While it has long been predicted that N-fixers can be distinguished from non-N-fixers using a relatively inexpensive assay for leaf nitrogen concentrations and δ^15^N^9^, this potential application is less routine, particularly in natural systems^10,11^ Virginia and Delwiche^12^ directly tested the assumption that isotopes predict N-fixing phenotypes in the field, comparing isotopic signatures of N-fixing and non-N-fixing taxa across a set of field sites in California that varied in habitat, soil chemistry and distance to the coast. The authors also took soil samples to determine soil chemistry, including soil δ^15^N. In sum Virginia and Delwiche^12^ found that low δ^15^N abundance, along with foliar N amount, “should serve as presumptive evidence of atmospheric N_2_-fixation.”

The importance of elemental stoichiometry in predicting N-fixing status has also been suggested^12^, but it may be particularly useful given recent plant physiology studies. Perhaps as a consequence of being able to utilize atmospheric N^13^, N-fixing plants have been shown to generally produce excess per-area leaf nitrogen^14^. This excess leaf nitrogen enhances intracellular CO_2_ use for photosynthesis through increased investment in the photosynthetic apparatus, the greatest N cost to plants, which ultimately limits water loss^14^, and is thought to contribute to the ability of N-fixing plants to thrive in arid environments^15,16^. N content of leaves is frequently studied in plant ecology and is available for many species^17^, but to our knowledge such data have yet to be quantitatively assessed with isotopic ratios for their potential to predict N-fixing status.

An isotopic and elemental approach could be used in the detection and measurement of nitrogen fixation on sites where direct recovery of nodules is difficult or impossible^12^. This possibility would be invaluable for elucidating the evolutionary history and geographic patterning of N-fixation and nodulation, particularly because it would enable the use of museum (herbarium) collections to permit the inference of N-fixing status for many species of currently unknown nodulation status, as nodules are often challenging to obtain in the field. Isotopic or elemental data could also be a proxy for nitrogen fixation rate^12^, which is important because some N-fixing plants are thought to be facultative in their fixing^18^.

The N-fixing ability of legumes has been assessed previously using the natural ^15^N abundance technique, primarily to quantify N-fixation in agroecosystems^10,11,19^. This information would also have value in natural systems, because many N-fixers are able to modulate their investment in nodules based on the environment, season, and developmental stage, thus changing their reliance on atmospheric N in response to ecological challenge^20,21^. Still, information on fixer status remains unavailable for all but a few species and ecosystems, largely because standard assays relying on direct detection of nitrogen fixation require complex experiments such as growth chamber setups or field manipulation of ^15^N^10,22^. A field-based, non-manipulative assay would be powerful and complementary to detailed experimental approaches in allowing many species to be assayed quickly in natural conditions and in a variety of geographic areas, ecological community contexts, and time points. Such an assay would have the additional benefit of simultaneously assessing leaf economic traits, which serve as proxies of leaf tissue quality^23,24^.

Here we extend work by^12^ in multiple ways, taking a similar broad and regional approach to field-collecting isotopic data but focusing on predictive ability to assess fixers versus non-fixers. In particular, rather than examine elemental and isotopic patterns across sites, we treat the identification of N-fixing phenotypes as a classification problem, in part. If we have isotopic data for a set of fixing and non-fixing plants at a site across a broad region with differing environments, and nothing else, can we predict nitrogen fixing status? We took this approach particularly to understand its applicability for potentially studying museum specimens of species for which N-fixing phenotypes have never been studied previously. Beyond classification of N-fixing plants, we also use our field study system to better understand how much phylogenetic context and environmental context, including soil conditions, together determine both isotopic signature ratios and per-area leaf carbon and nitrogen.

We implement a field collecting design across a portion of the USA Southeast and specifically ask: (1) with what accuracy can N-fixing status be predicted using leaf chemistry data, irrespective of variation in habitat type, taxon, soil chemistry, native status, and other biotic and abiotic variables? We also ask: (2) whether abiotic environmental and habitat impact our ability to predict N-fixing phenotype and (3) if there is strong phylogenetic signal in isotopic signatures and foliar chemistry. Finally, we explicitly opted to sample exotic (non-native) fixers as well as native ones and anticipated that many exotic fixers may have been intentionally introduced because of their greater fixing capacity. We thus ask whether (4) exotic N-fixers would potentially show lower δ^15^N signatures, as well as other compositional differences, compared to native N-fixers. In sum, our work broadly addresses multiple key questions on the drivers and predictive capacity of leaf foliar chemistry in relation to N-fixing phenotypes. This effort can serve as a starting point for developing model-based predictive approaches for using strategically collected field and eventually museum samples to make predictions more broadly.

## Methods

### Study sites and field design

We developed a field design that maximized our ability to derive insights across sites, species, and environments by replication at each level. Sites were defined as small plots under 100 m^2^. Plot selection was first based on representation of major habitat types in the study area and then prospective sites were assessed for the presence of focal taxa. At least four focal taxa outside of the N-fixing clade (NFC) and two known N-fixers based on Kates et al.^2^ needed to be present for a site to be selected. Presence of other nearby habitat types was also taken into consideration during site selection. When possible, sites representing different habitat types were selected in close proximity to one another to limit spatial variation that was not due to habitat differences; conversely, replicate habitat sampling was done at more distant sites to reduce spatial autocorrelation. The recognized habitat types, from^25^, included here are: closed canopy forest, mixed forest, pine flatwoods, upland sandhill, maritime hammock, cypress swamp, saltmarsh, prairie, and disturbed areas. Most collections were performed during peak green in the summer of 2021. Table 1 summarizes habitat type and number of sites per habitat, along with sampling rates for fixing and non-fixing species.

**Table 1:** Summary of sampling across habitat types and sites in Florida, Alabama and Mississippi.

Habitat Type	# of Sites	# legume N-fixers species	# legume N-fixers samples	#non-legume N-fixer species	# non-legume N-fixer samples	#non-N-fixer species	#non-N-fixer samples
CC forest	2	7	13	0	0	7	8
Disturbed	8	21	41	2	2	17	24
Flatwoods	2	6	13	1	3	8	10
Maritime	2	5	12	1	2	10	10
Mixed	4	12	28	1	2	12	21
Prairie	1	7	12	0	0	2	2
Saltmarsh	1	3	5	0	0	5	5
Swamp	1	1	2	1	1	8	8
Upland	6	18	53	0	0	21	30

Most habitat types had replicated site sampling and all sites had both known fixers and non-fixers sampled. See Methods and Supplementary Table S1 for further site sampling details.

### Sampling design

**Figure 1:**
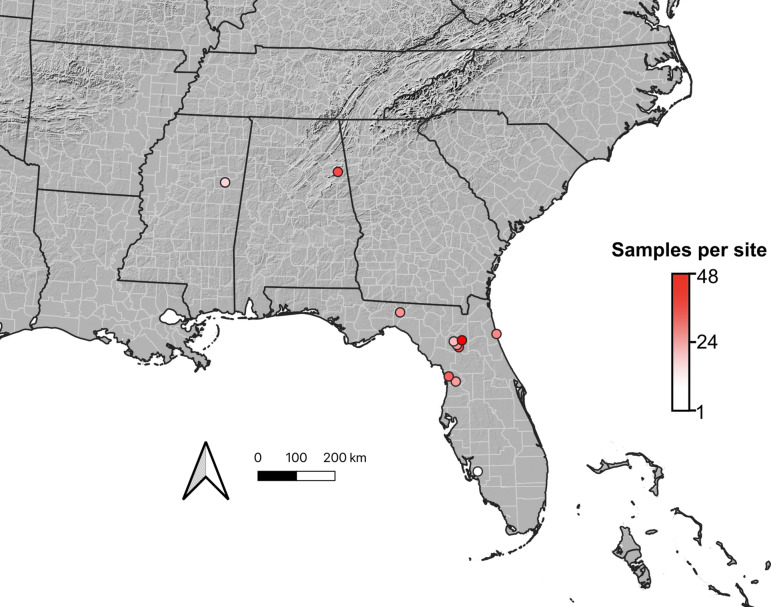
Map of field sites in the southeastern United States for leaf material sampling. Projection: NAD83 (EPSG:26960). Figure created in R version 4.3.1.

We implemented a structured taxonomic sampling design at each study site to cover a diversity of both N-fixing and non-N-fixing species. The non-N-fixing specimens, included as references representing plant material whose N derives only from soil N, were selected based on their phylogenetic position relative to the nitrogen fixing clade and likelihood of presence across sites within our study area (Fig. 1)^23,26,27^. These taxa included: *Quercus* (Fagaceae) and *Rubus* (Rosaceae), two common genera across the southeastern U.S. that are non-N-fixers within the Nitrogen-Fixing Clade (NFC; two *Quercus* species were sampled from each site when two were present)^28,29^; Asteraceae, a common eudicot family across the focal area well outside of the NFC; and Poaceae, a common monocot family within the study area also well outside the NFC. Lastly, a gymnosperm was selected—a member of Pinales, in nearly all cases a member of *Pinus,* representing the non-N-fixing taxon most distantly removed from the nitrogen fixing clade.

For known N-fixers we verified the presence of nodules where possible (see below) and collected mature leaves and vouchers (deposited at FLAS) from two individuals of every species present at the site, assuring sampling replication for most N-fixing taxa within and across sites (Table 1). Although species-by-site level replication is limited, we note that many species were collected across sites, including sites from the same habitats, thus supporting broader habitat replication. As our primary benchmark, we assessed N-fixing traits for the purpose of sampling and model fitting by following a recently reported up-to-date nodulation database^2^. Kates et al.^2^ collated determinations of nodulation status derived from high-quality reports of nodulation and expert consultation, considering available field and experimental data as well as phylogenetic relationships. Due to limited data at the species level, these data were scored at the genus level. Genus-level nodulation determinations are established practice for large-scale studies^2,16,30–33^, and while species-level observations are ideal a higher-level taxonomic determination is justified by the uniformity of N-fixing phenotypes in many large clades^34^. During vouchering all specimens were inspected for nodules and these presences were confirmed for the majority of known N-fixers included here. Unfortunately, nodule absence is more challenging to confirm due to soil type, root depth, and other collection factors as well as seasonal variation in nodules. Finally, we were unable to collect all root material for some individuals due to permitting restrictions, which also impacted assessment of nodules for a minority of samples. However, all field observations of nodules were in line with our expectation based on the nodulation database.

The N-fixers are represented by three families: Fabaceae, Myricaceae, and Elaeagnaceae; and within Fabaceae, by 13 tribes sensu^35^. Fabaceae (the legumes) comprise the vast majority of the species in the study area and are characterized by rhizobial symbiosis (symbiosis involving alpha- or betaproteobacteria), and which in the study area are thought to be nodulated primarily by members of alphaproteobacteria^36–38^. Myricaceae (represented by two species of *Morella*) and Elaeagnaceae (only represented by the invasive *Elaeagnus pungens*) are instead characterized by actinorhizal symbiosis, where the plants are nodulated by actinobacteria in the genus *Frankia*^38–41^.

Ultimately, we sampled mature leaves and took vouchers from a total of 305 individual plants from 25 sites, with 171 of those samples being from species that are known N-fixers (Fig. 1; Table 1). Although species-by-site level replication is limited, we note that many species were collected across sites, including sites from the same habitats, thus supporting broader habitat replication. Rhizobial and actinorhizal symbioses occur in distinct plant lineages^34^; the plants and symbionts differ anatomically and physiologically^38,40,42^ as well as in global geographic distributions^33,39–41^. For these reasons, we examined N-fixing predictions across all taxa.

### Exotic plants

Because all N-fixer taxa present at a site were collected, we included a combination of both native and exotic species. Exotic status followed the USDA PLANTS database determinations of invasive and noxious status (https://plants.usda.gov/home/noxiousInvasiveSearch) and cross-referenced with native/nonnative determinations from Plants of the World online (POWO; https://powo.science.kew.org/). Most exotic species included are within genera that also have native representatives (e.g., *Crotalaria*, *Desmodium, Indigofera*). There are, however, taxa that do not occur natively in the study region (family Elaeagnaceae and the legume genera *Arachis, Alysicarpus, Albizia,* and *Melilotis*) or were only represented in the region of interest by exotic species (*Trifolium*). We did not sample sites with legally controlled noxious weeds, thus we focus our comparison on natives vs. exotics rather than assessments of invasiveness.

### Soils

Two 50 ml soil samples were taken from each site with a spade after removing the organic layer (generally leaf litter). This depth (standardized at 15 cm) is consistent with that of the rhizosphere of the herbaceous N-fixers collected at the sites. Where there were differences in elevation or hydroperiod within a site, the two samples were taken at each extremum. When the sites were homogenous, the two samples were taken on opposite sides of the site. Soil samples were frozen after collection and then homogenized using a mortar and pestle that was cleaned with ethanol between samples. The homogenized soils were then lyophilized for ~ 4–5 days. Freeze-dried samples were then loaded into tin capsules, with sample mass for sandy soils around 25 mg, carbon-poor soils around 10 mg, and carbon-rich soils around 5 mg. These soil samples were then analyzed for wtC, wtN, δ^15^N, and δ^13^C using a Carlo Erba NA 1500 CNS Elemental Analyzer with A ConFlo III interface linked to a Delta V isotope ratio mass spectrometer (IRMS).

### wtN, wtC, and isotopic analysis

Leaf samples were taken from each plant in the field before vouchers were pressed. The leaves were placed in paper envelopes and covered with silica powder for desiccation. Leaf samples were then packed into tin capsules containing 1.0 to 1.5 mg of leaf material. Each leaf sample was analyzed for wt wtC, wt wtN, δ^15^N, and δ^13^C at the Light Stable Isotope Mass Spec Lab at the University of Florida, Department of Geological Sciences. Isotopes ratios were calculated as [δ^13^C, δ^15^N] = [(R_sample_/R_standard_)−1] × 1000, where R_sample_ and R_standard_ are the ratios of heavy to light isotope (^13^C/^12^C and ^15^N/^14^N) in the sample and international standard, respectively. The international standard used for ^13^C was Vienna Pee Dee Belemnite and for ^15^N was AIR (Ambient Inhalable Reservoir). The analytical accuracy of isotopic measurements was calculated as the SD of replicates of working standards USGS40, USGS41a, and wheat flour (in supplemental).

### Modeling framework: phenotypes

To address our primary question as to whether we can use wtC, wtN, δ^15^N, and δ^13^C to predict the nitrogen fixing status of an individual plant, we used the R package InformationValue^43^ to implement a classification model. We assigned random subsets of 75% for the training dataset and the remaining 25% of samples for the unknown test set, performing this procedure in 10 replicates. We then used the training dataset to calibrate a logistic regression model predicting an N-fixing presence/absence response using wtC, wtN, δ^15^N, and δ^13^C values as predictors, with habitat treated as a random effect. This model was of the form: N-fixing ~ δ^13^C + wtC + δ^15^N + wtN + (1 | habitat). Predictor importance in the model was assessed using standardized coefficients. Given that fixer status for each species is already known, we can then use the predict function in the R package ISLR^44^ to assess probability of N-fixing phenotypes for our test data, and threshold models using function optimalCutoff to make a yes/no prediction for N-fixing phenotypes. To obtain the predictive power and the uncertainty of the model, we did the same approach for each of the 10 replicates. We then used ISLR^44^ to create a confusion matrix, which quantifies the number and type of classification errors made by the model compared to known fixing status.

### Model selection: abiotic environment

The impact of abiotic variables on the predictive power of the predictors used above (wtC, wtN, δ^15^N, and δ^13^C; here treated as the response) was assessed using generalized linear mixed models (GLMM). The rationale for the use of a mixed modeling approach was to separately partition and assess variance in the elemental and isotopic data explainable by abiotic environment (fixed effects) and by spatial variation independent of abiotic environment (random effects). We fit multiple statistical models that only differed in the response variable used among the set: wtC, wtN, δ^15^N, and δ^13^C. First, we fit models with these four responses, with habitat treated as the fixed effect and genus as the random effect. Then, to account for soil effects, we fit a set of models treating soil wtN and wtC as fixed effects and genus as the random effect.

After fitting the full model, we used the R package MuMIn^45^ to examine all subsets of fixed effect terms in the global model, and select the best model predictor set via AIC. As our main purpose in this model set was to investigate potential key drivers through hypothesis testing instead of phenotype prediction and because second-best models were > ΔAIC 2, we did not conduct model averaging. After model selection, we checked all final best models for variance inflation (VIF) using the vif function in the car package^46^, and found that VIFs for models were never above 5, confirming limited multicollinearity among our predictors.

### Model selection: phylogenetic signal

Several studies have shown that elemental stoichiometry of leaves is shaped by phylogenetic conservatism^23,47^. To estimate the phylogenetic effect of elemental and isotopic data as opposed to N-fixation effect, we calculated phylogenetic signal using covariance as implemented in R package phyr^48^. The phylogeny of species across the sites was generated using R package rtrees^49^ based on a recent synthetic dated phylogenetic tree spanning the seed plants^50^. This phylogenetic approach allows for mixed models comparable to the main analysis and thus a direct estimate of the relative contribution of phenotype and phylogeny that is not detected by standard phylogenetic signal tests. Four models were fit for each of the elemental and isotopic datasets, with N-fixing as the fixed effect and phylogeny and habitat as random effects. To investigate the specific contribution of phylogeny, models with and without a phylogenetic term were compared by AIC.

### Model selection: exotic status and N-fixing phenotypes

Determinations of N-fixing phenotypes via elemental and isotopic data can be complicated by life-history variation,^23,51,52^ and can exhibit plastic responses in some environments^53^. Based on the inclusion of many exotic N-fixers in the study region and the expectation that exotic N-fixers are enriched for weedy traits, or potentially were selected for N-fixing ability for use in agriculture, we expected exotic species to exist on a different part of the leaf economic spectrum^54,55^ compared to natives. We tested this by treating each of the four isotopic/elemental datasets as responses in separate models, fitting to each native status as the fixed effect and both habitat and N-fixing as random effects. The latter two were included because exotic taxa in this dataset overrepresent N-fixers and ruderal habitats, but native status independent of these effects was the quantity of interest.

## Results

### Prediction of N-fixing phenotypes

**Figure 2:**
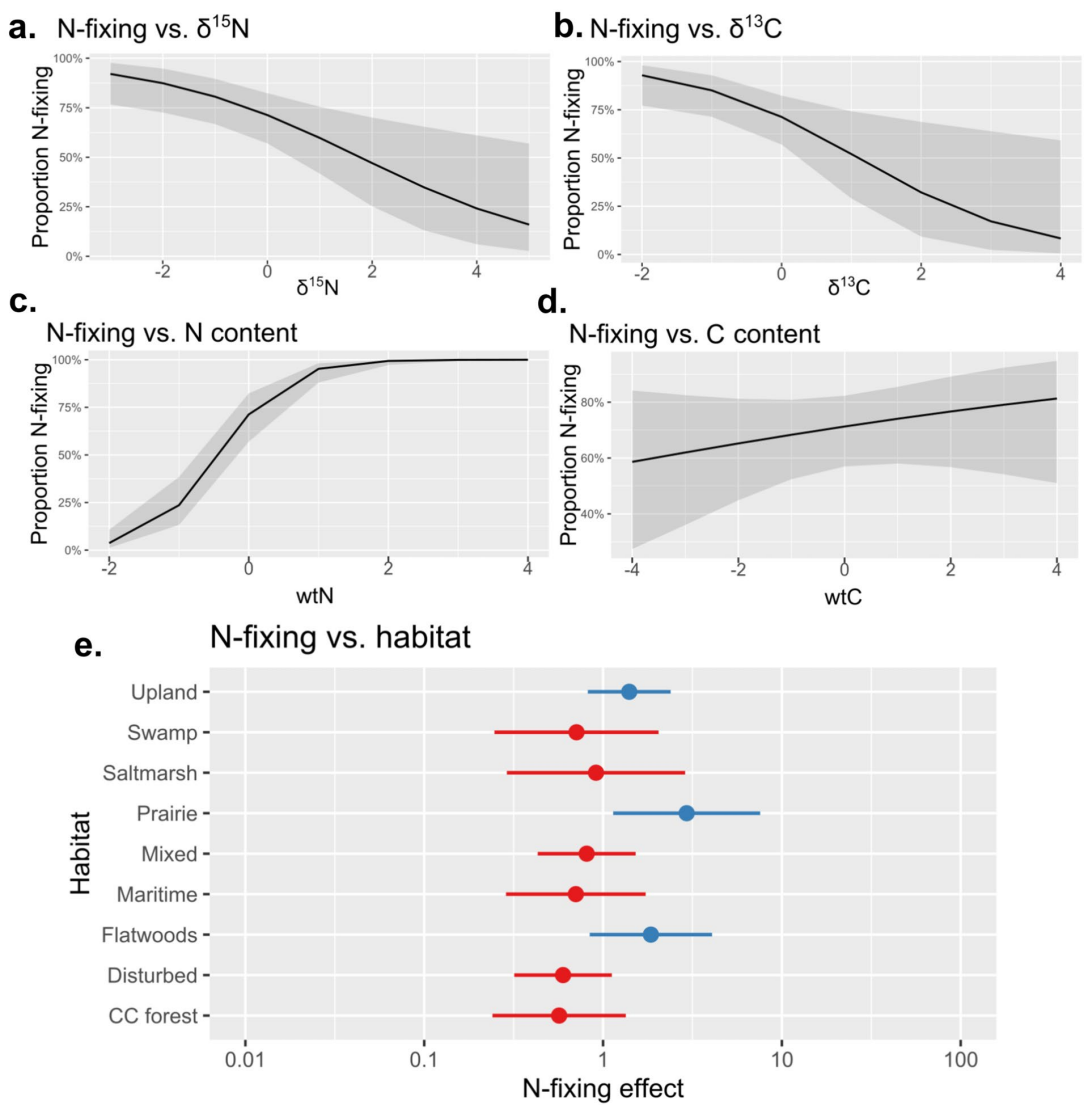
Standardized effect plots for the main prediction model, where (**a**–**d**) are the fixed effects (gray curve represents the 95% confidence interval) and (**e**) is the random effect (blue effects are positive, red effects negative). Figure created in R version 4.3.1.

The first question we tested was: can we use elemental and isotopic signatures to predict nitrogen fixing phenotypes? The model with all elemental and isotopic predictors was favored, with conditional *R*^2^ 0.695 and marginal *R*^2^ 0.651, indicating a strong fit overall with most of the phenotypic variance explained in the fixed effects (elemental and isotopic data, rather than habitat). The predictive modeling approach determined that wtC, wtN, δ^13^C, and δ^15^N together predict N-fixing phenotypes with cross-fold validation yielding 80.3–93.6% accuracy. The prediction model was strong overall based on Area Under the Curve (AUC) metrics on the test data, often used to assess model performance (0.8238–0.9362). Sensitivity in the prediction (0.7451–0.9818 in the 10 replicates) was higher than specificity (0.6896552–0.92), which indicates that the prediction models made relatively few Type I errors (false positives of N-fixation), and somewhat more Type II errors (false negatives). Figure 2a–d shows model effect plots for all 4 predictors and demonstrates the importance of wtN as a key predictor of fixing status.

**Figure 3:**
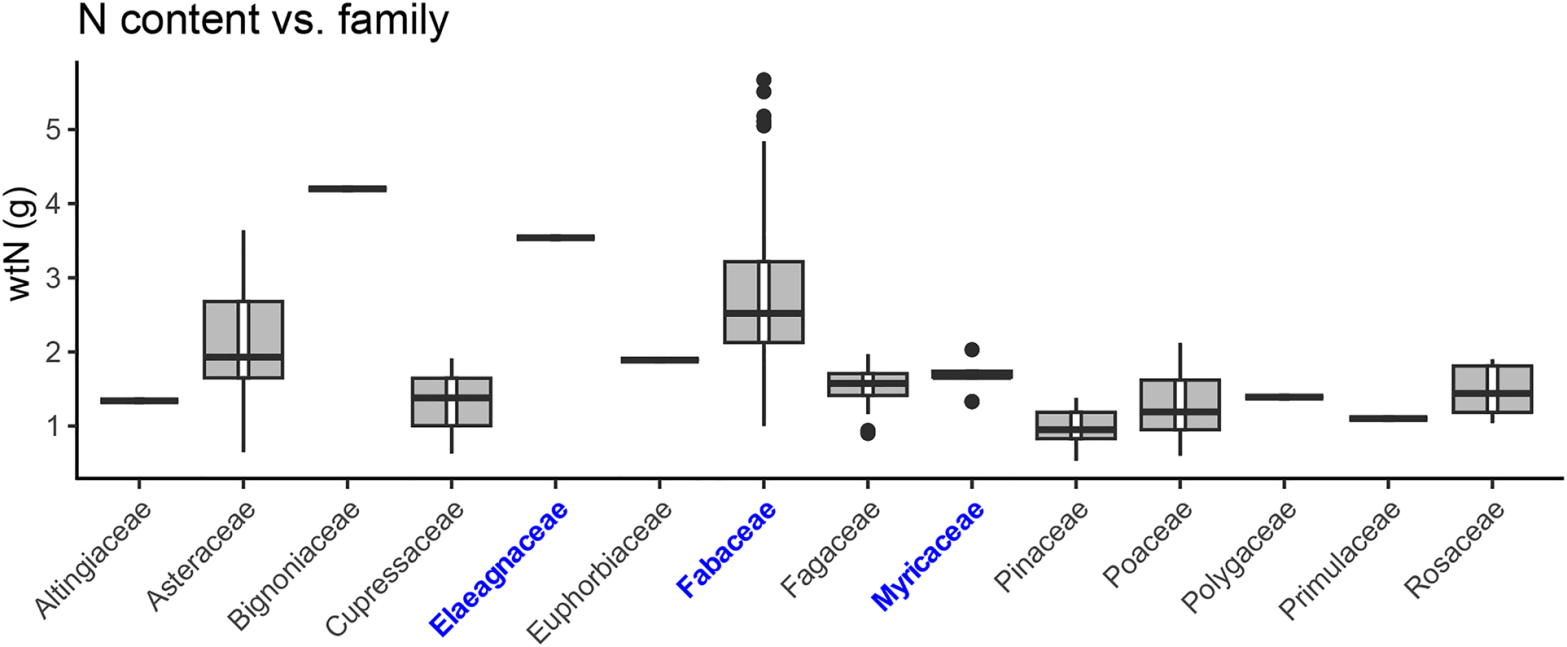
Boxplot of raw wtN data by family. Families in blue/boldface contain known N-fixers collected in this study. Note that N-fixers, especially legumes (Fabaceae) are usually higher in wtN, but two outlier families (Bignoniaceae and Asteraceae) also are relatively high in wt wtN. Figure created in R version 4.3.1.

Surprisingly, the most predictive variable was wtN, not δ^15^N; wtN importance was greater than the next-best predictor (δ^13^C) by more than two-fold and four-fold greater than δ^15^N on the basis of standardized coefficients. Hence the percent nitrogen in leaves of N-fixers is highly predictive of N-fixing phenotypes; nitrogen isotope content separately contributes important information to the classification but by itself contains less information than nitrogen content. Figure 3 shows Fabaceae in general has higher weight N compared to other non-fixing groups and some other fixing lineages (See Supplementary Fig. S6 for other variables).

### Impact of abiotic environment

The second question we tested was: does abiotic environment impact our ability to predict N-fixing phenotypes? We fit models including habitat and soil elemental data as predictors, and the elemental and isotopic data of plants as the response, to investigate whether the measured traits were significantly affected by environmental parameters, in each case separately partitioning variation due to taxon (here, genus-level). Stepwise model selection did favor the inclusion of habitat in the model for the most important predictor (N content), but habitat explained little of the variance independent of taxon (conditional *R*^2^ = 0.581 and marginal *R*^2^ = 0.047, meaning most of the variance was in the random effect; results were similar for C content and both isotope responses with all marginal *R*^2^ < 0.05; see Supplemental Figs. S1–S5). Soil nutrients also had a limited effect on leaf isotopic and elemental content. Leaf wtN content and δ^15^N were completely unaffected by soil, while soil N was a significant model term for δ^13^C (*p* = 0.00977) and soil C was significant for wtC (*p* = 0.032). Hence, the most important predictors for the N-fixing model were unaffected by soil environments; similarly while the N-fixing model predictors differed by habitat, habitat explains little of the variance compared to taxonomic identity (which in this dataset effectively includes variance due to N-fixing). As noted above, the primary N-fixing prediction model also contained habitat as a random effect, and in this context only the prairie habitat had a significant effect on N-fixers (Fig. 2e).

### Phylogenetic structure in isotope and element analysis

**Figure 4:**
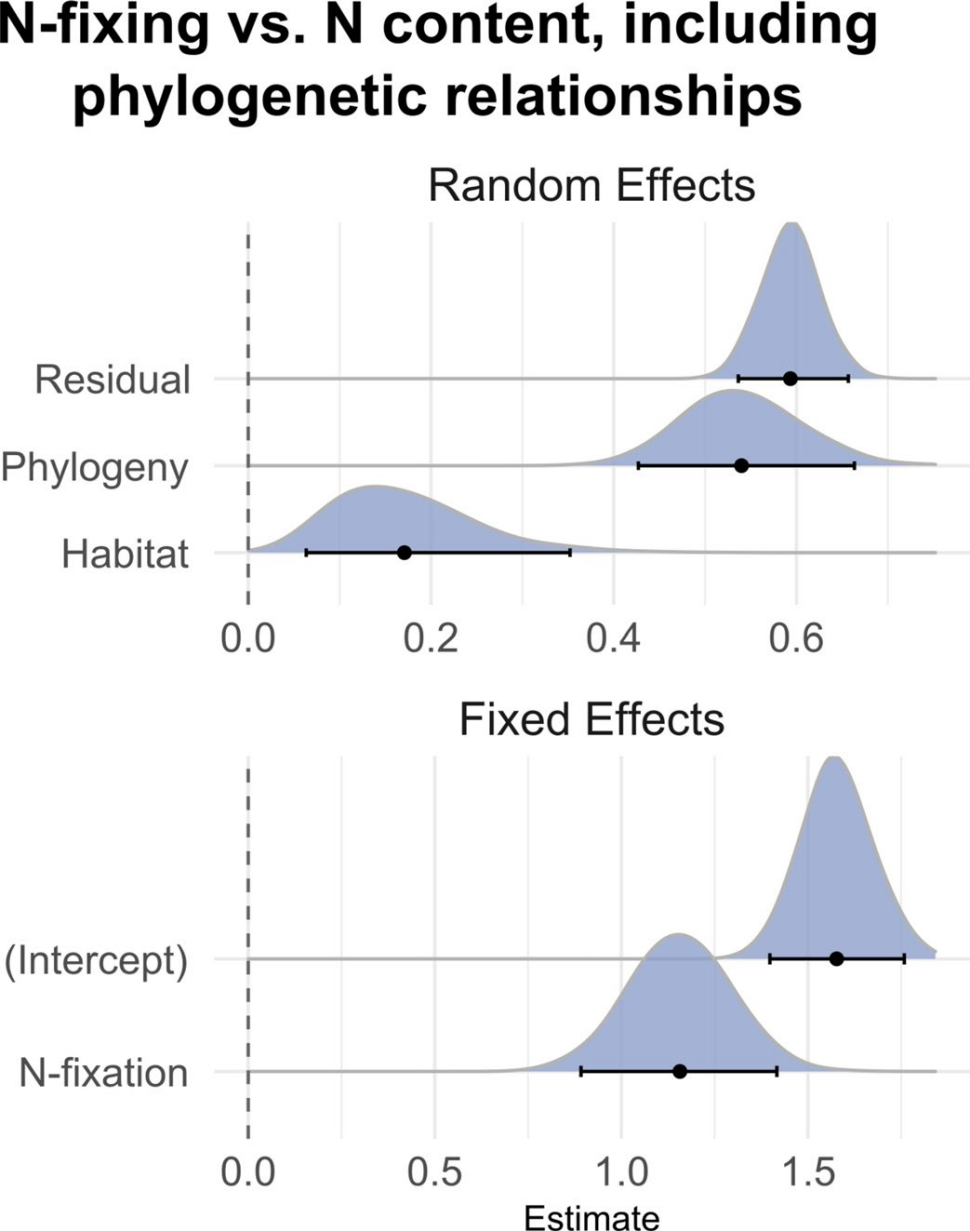
Effect plot for phylogenetic model, here showing wtN as the response. Figure created in R version 4.3.1.

Our third question: are isotopic and are leaf chemical traits directly structured by phylogenetic relationships? To test this, we fit N-fixing as the fixed effect (to separate variance due to the N-fixing phenotype), and random effects for habitat and phylogeny. All four models strongly favored inclusion of phylogeny as a random effect (ΔAIC > 13 in all cases); in all models except that with wtC as the response, N-fixing was a significant effect, mirroring the results in the predictive model. Thus, phylogeny shapes all four responses, but N-fixing phenotypes show a highly significant response as well that is independent of phylogeny and nearly twice as strong; the contribution of habitat was significant but marginal by comparison (Fig. 4).

### Exotic species and element/isotope composition

**Figure 5:**
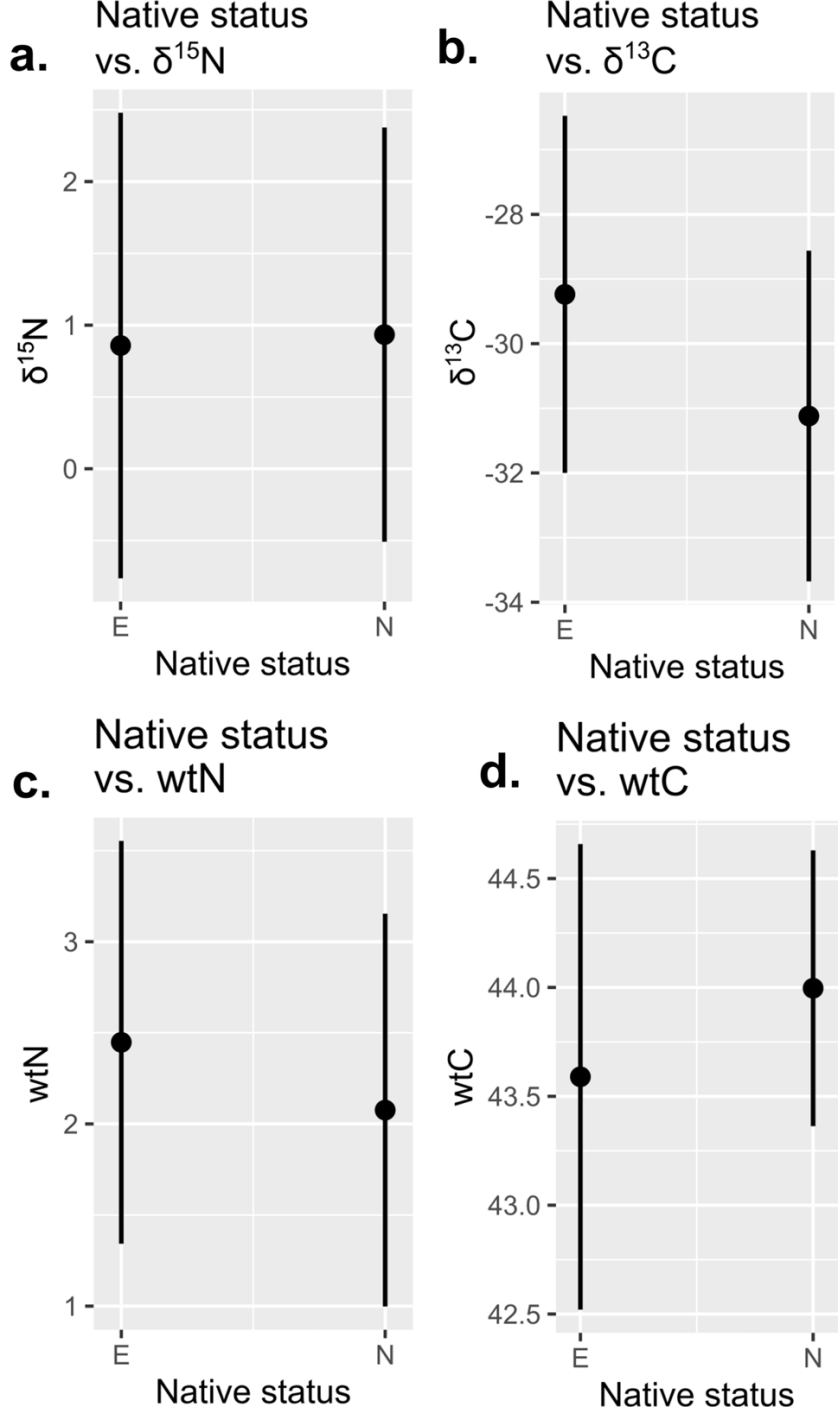
Fixed effect plots for effect of native status on elemental/isotope data, with both habitat and N-fixing status treated as random effects. (**a**–**d**) are different models for each data type. Note only δ^13^C and wtN (**b** and **c**, respectively) were significant and the effect size was modest. Figure created in R version 4.3.1.

The final question we investigated was: do native and exotic N-fixers vary in their elemental and isotopic signatures? Linear mixed models, treating habitat and N-fixing status as random effects, demonstrated that, while the fixed effect was modest, exotic taxa had significantly higher leaf wtN (*p* = 0.0113) and δ^13^C (*p* = 0.00288) compared to native taxa (leaf C content and δ^15^N were non-significant; Fig. 5). Because certain genera in the dataset are enriched for exotic taxa, we also constructed a set of models fitting both phylogeny and N-fixing as random effects. In this model set, only wtN was significant (*p* = 0.001434). Thus, exotics are enriched for N as previously observed^55^, and this effect is independent of habitat, phylogeny, and N-fixing status.

## Discussion

We demonstrate here that N-fixing phenotypes can be predicted for phenotypically unknown taxa. We achieved relatively strong predictions of N-fixing phenotypes, but this required the use of multiple predictors jointly for the best classification success. A combination of δ^13^C, and δ^15^N, wtC, and wtN provided the best metric for determining the N-fixing phenotype, with an accuracy of 80–94%, and the strongest contribution from wtN. Often, nodulation status is determined by phylogenetic relatedness, rather than any empirical nodule evidence, even if that evidence is indirect^31,56^. This is often due to the labor intensiveness and impracticality of observing roots and nodules in situ. The rapid and inexpensive assay and modeling approach we report suggests a path to overcoming the shortage of taxa that have been studied for symbiotic states, although more work to gather synoptic data across sites broadly may still be needed. This assay can also be performed on museum samples, preferably if there are multiple collections of candidate potential fixers and non-fixers at the same locality. We argue that combining foliar measurements and phylogenetic information is likely to be highly reliable for predicting fixer status when used in combination. The phylogenetic conservatism of foliar chemistry suggests that distinguishing N-fixing states within or among closely related species should be particularly successful.

### Model performance

We found that our predictive model was more likely to make errors of omission (false negatives) than commission. This is a desirably conservative property because discoveries of unknown N-fixers would be the finding of interest in this context; negative findings could reflect an N-fixation phenotype that was not recovered, but this is less problematic than false discoveries of N-fixation^57^. However, this also implies that negative reports have somewhat less weight and a single observation will not completely rule out the actual lack of the nodulation phenotype. Remaining uncertainty could be improved by more field replication, particularly including multiple seasonal time points (see below). Related to this, despite a wide range of abiotic factors, none had a significant impact on our ability to determine the N-fixing phenotype, hence soil and other environmental factors are unlikely to be behind misclassification.

A more likely explanation, also problematic for direct field observations of nodulation^56^, is the effect of developmental stage. Nodules are known to be highly seasonal organs in many taxa^58,59^ samples from plants that are less physiologically active or senescing may represent an important confounding variable to control in the future. As well as nodule seasonality, leaf senescence is also thought to have direct effects on δ^15^N^12,60^, underlining the importance of controlling for or including developmental stage effects.

A final likely reason for misclassification is the presence of high leaf N in two included non-N-fixing families (Asteraceae and Bignoniaceae), likely representing phylogenetic structuring of leaf N^23,61^. Considering only taxa within the nitrogen-fixing clade, most likely to contain unknown N-fixing phenotypes, would remove variance due to N variation in distantly related lineages. In general, a smaller taxonomic scope would likely improve prediction success by controlling phylogenetic variation in leaf elemental composition. We note that the mechanisms that might support higher leaf nitrogen proportions in Asteraceae and Bignoniaceae compared to other angiosperms^61^ and other high-N non-N-fixing taxa are not completely known. Thus, these assays are not only useful in predicting symbiotic N-fixing phenotypes, but generating data-driven insights about other plant clades that can be more fully explored.

### Best N-fixation predictors

A surprise in our prediction model concerns the relative contribution of the predictors, with N content being the most important. Leaf nitrogen has long been known to be elevated in N-fixing plants^13,62,63^ and this contrast is robust to spatial and ecological context^14^ and likewise true of disparate lineages of N-fixing plants^13^. Rather than a direct consequence of N-fixation, high N content in N-fixing plants likely reflects ecological specialization of N-fixers with rich leaf N that is facilitated by symbiosis^13^, particularly their characteristic predilection for open and relatively dry habitats^14^. Regardless of the physiological mechanisms behind leaf elemental content, as our study shows, this information provides for high quality phenotype predictions.

While δ^15^N was also an important predictor in the model, its importance was secondary (Table 1); this result was robust to inclusion of habitat and soil N in the models. A reason that δ^15^N was not the top predictor may lie in differences in nitrogen fixation rates among the included plants. N-fixation and nodule production differs seasonally^20,21^, in response to soil N availability^64–67^, and between rhizobial and actinorhizal forms of symbiosis^38,42^. This finding suggests a second use of isotopic data as a proxy for N-fixation rates as has been investigated previously^10,19,60^. The differences between legumes and non-legume N-fixers, for instance, may relate to differences in N-fixation rates between forms of the symbiosis^38,42^. However, there are far fewer actinorhizal than rhizobial taxa in the study area, so further sampling within taxa may be needed to fully characterize the range of isotopic and leaf stoichiometric values in actinorhizal plants. 

The second-best predictor in our model was δ^13^C; the reason for this predictive value likely relates to the significance of N-fixation for photosynthetic physiology. δ^13^C is increased under higher water use efficiency due to higher intracellular sourcing of CO_2_^68,69^, and accordingly these results agree with the finding of^69^ that high leaf δ^13^C is associated with high wtN. While^69^ found that δ^13^C scales with wtN regardless of N-fixing status, arguing this was due to shared photosynthetic effects, our classification models found independent predictive value for δ^13^C. The already noted connection between photosynthetic physiology and N-fixing symbiosis suggests the reason for the importance of δ^13^C; leaf nitrogen allocation is closely related to carbon assimilation^14,70^. Alternative photosynthetic pathways such as CAM and C_4_ can also modify δ^13^C^71^, but these are unknown in the N-fixers studied here. However, differing photosynthetic parameters such as photosynthetic rate and developmental stage also affect δ^13^C^72,73^ (Table 2).

**Table 2:** Best model for phenotyping: predictor importance and significance.

	Standardized coefficient	Pr( >|z|)
δ^15^N	− 0.5137	**0.00591**
δ^13^C	− 0.828	**0.0124**
wtN	2.0845	**4.18E−12**
wtC	0.1402	0.36982

wtC was the only non-significant predictor and had the least effect; wtN had more than twice the effect of the next-best predictor (δ^13^C).

Significant values are in bold.

### Nonnative effects

The significant effect of native status on wtN and δ^13^C, rather than δ^15^N, was unanticipated. However, elevated leaf nitrogen has been observed before in exotic taxa^55^. We investigated this further to see if this reflected an interaction between native status and phylogeny, finding that only the wtN result is robust to the effect of phylogeny. Thus, while exotic taxa are a phylogenetically concentrated subset of the larger N-fixing clade, high leaf nitrogen is characteristic of exotics even when this is taken into account. This finding may reflect the ecological strategies of exotic N-fixers; nonnative plants tend to exhibit weedy traits and occupy distinctive leaf economic spectra^54,55^. However, the impact of native status was small (Fig. 5c) compared to N-fixation itself, indicating that isotopic and elemental signatures effectively identify N-fixing phenotypes in a variety of ecological contexts.

## Conclusions

Here we have shown that N-fixing symbiotic phenotype can be robustly predicted using a rapid and inexpensive assay with traditional field collection. We built upon previous work by demonstrating the value of multiple predictors, and specifically the importance of leaf N content, which has not been used previously^60^. Integration of these in a classification model generates robust interpretation and may overcome difficulties associated with singular values of δ^15^N that have beset previous efforts^60^. The independent contributions of multiple predictors also means that we do not recommend the use of simple cutoff values (e.g.,^74^) and recommend future investigators also employ model-based strategies for phenotyping.

As noted above, replication (particularly temporally) as well as appropriate controls of known non-N-fixers and phylogenetic effects, increases the robustness of determinations. As argued by^57^, novel reports of N-fixing symbiosis, particularly outside the legumes, should be held to a high evidential standard. While this regional study design cannot yet be used to scale predictions too broadly without further efforts, our work provides a start at capturing relevant environmental gradients, and could serve for predicting fixer status from specimens, such as might be in natural history collections, from the same or similar regions or environments. A model-based approach such as that we propose here, especially if extended across more environments, will be important for screening naturally occurring species to generate strong candidates for validation using more detailed experiments, particularly for rare plants or areas of difficult field access. Despite recent databasing efforts^31,32,34^, our knowledge of N-fixing symbiotic states remains piecemeal and covers a limited portion of the species-level diversity^56^. Even fewer species have been studied for developmental, seasonal, and environmental variation in N-fixing status^18,20^. Assays such as those proposed here will be an important part of the toolkit for generating N-fixing phenotype determinations at scale.

### Supplementary Information

Supplementary Information.

## Data Availability

Raw data and analysis scripts are available via GitHub at https://github.com/ryanafolk/isotope. A stable release of this repository is available at: 10.5281/zenodo.8407949. all isotope data is located in our gtihub repo here: https://github.com/ryanafolk/isotope/blob/main/Nitfix_Isotopes_all_2N_fixed.csv. https://github.com/ryanafolk/isotope/blob/main/isotope_tree.tre“ and our synthesis phylogeny used for analyses is here: https://github.com/ryanafolk/isotope/blob/main/Nitfix_Isotopes_all_2N_fixed.csvhttps://github.com/ryanafolk/isotope/blob/main/isotope_tree.tre
