# Supplementary material for: Elemental and isotopic analysis of leaves predicts nitrogen-fixing phenotypes
Source: Sci Rep. 2024 Aug 29;14:20065. doi: 10.1038/s41598-024-70412-8 (PMC11362558; doi:10.1038/s41598-024-70412-8)
Supplement: Supplementary file 1 — Supplementary Information. [file 41598_2024_70412_MOESM1_ESM.docx]

Supplement to: Elemental and isotopic analysis of leaves predicts nitrogen-fixing phenotypes

**Supplementary Fig. S1.** Fixed effect plots for abiotic environment models, with δ^15^N (A), δ¹³C (B), wtN (C), and wtC (D) as responses and habitat as the fixed effect.


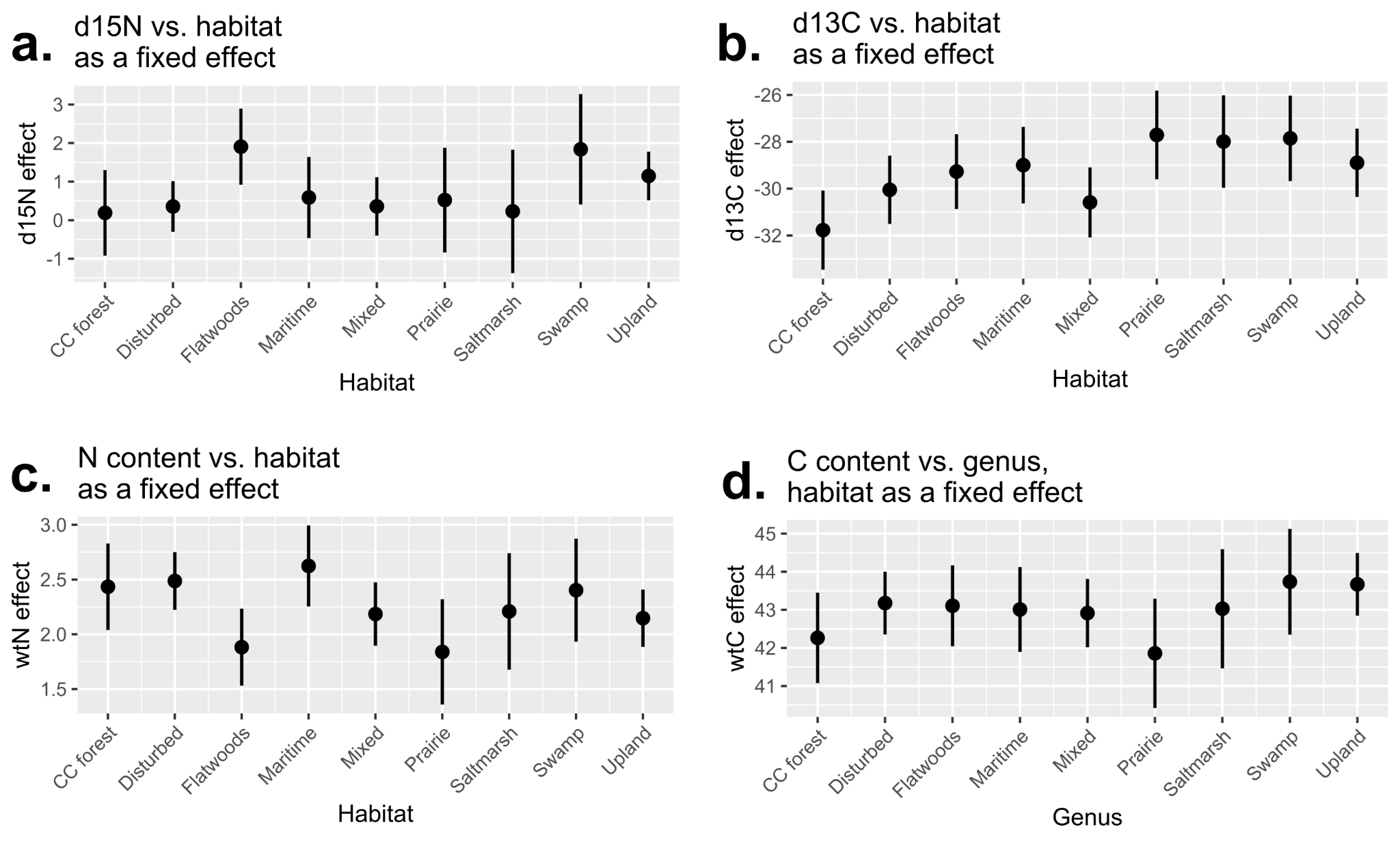


**Supplementary Fig. S2.** Random effect plot for the δ^15^N abiotic environment model, with δ^15^N as the response and genus as the random effect. (A), δ¹³C (B), wtN (C), and wtC (D) as responses and habitat as the fixed effect.


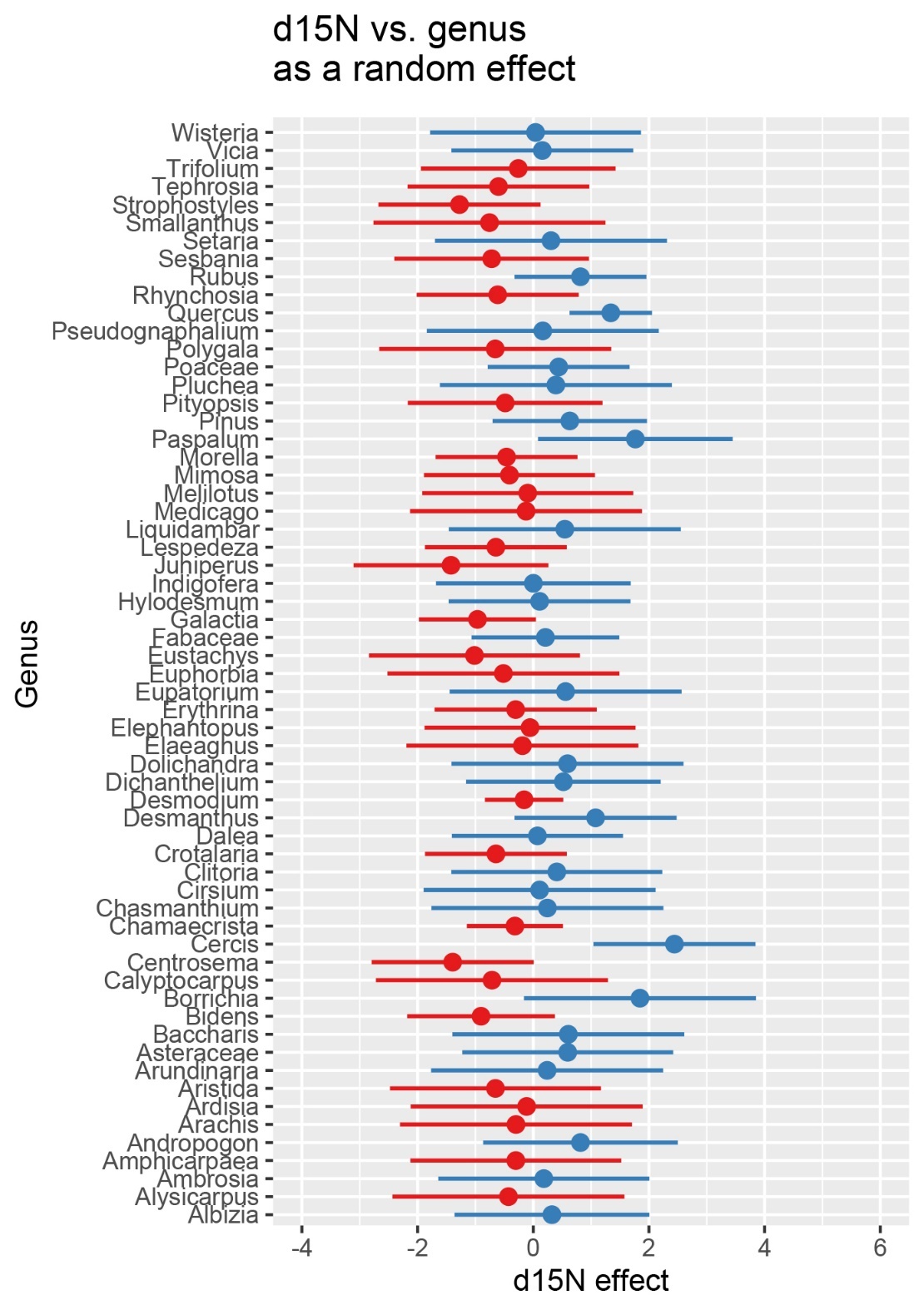


**Supplementary Fig. S3.** Random effect plot for the δ¹³C abiotic environment model, with δ¹³C as the response and genus as the random effect.


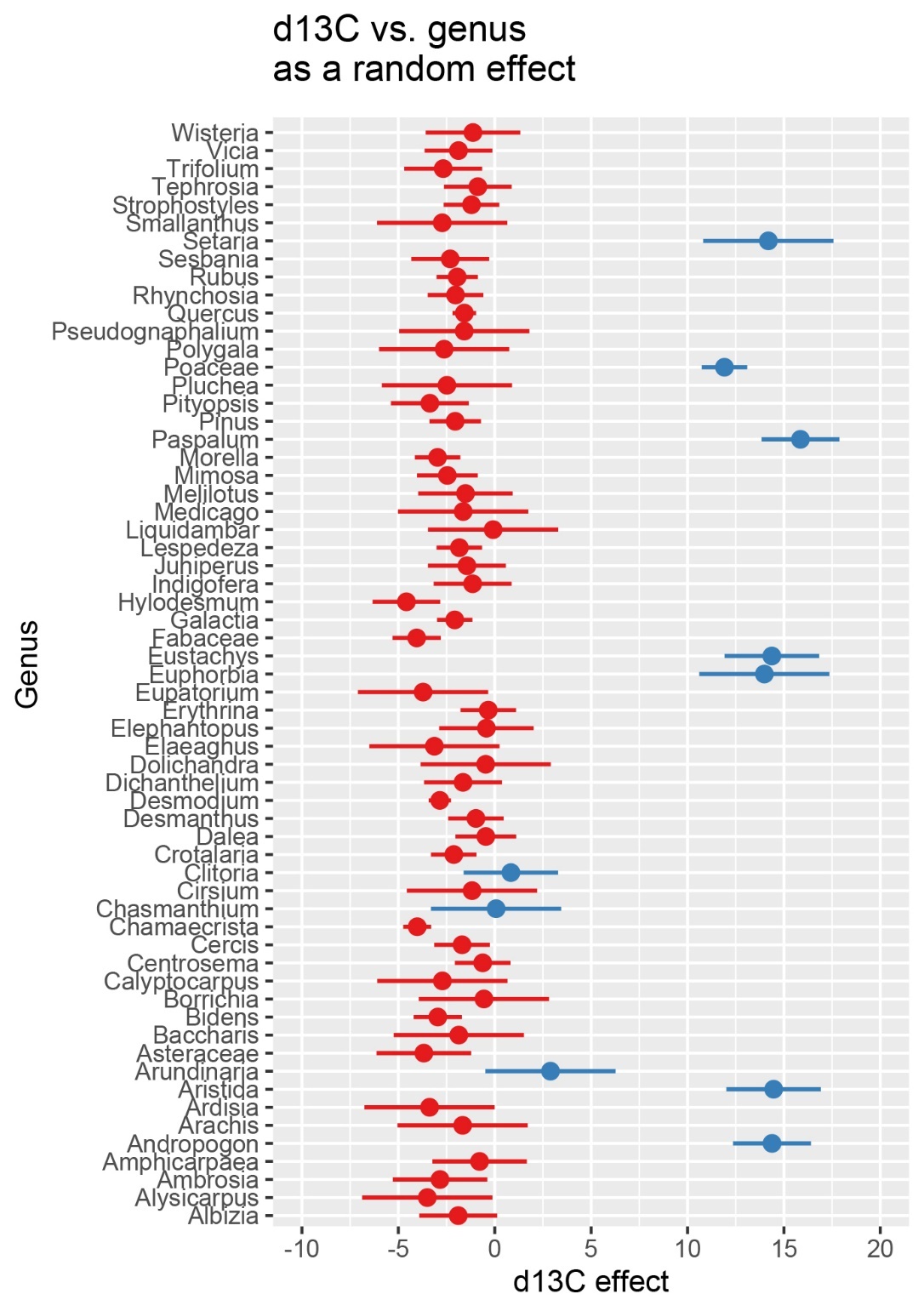
**Supplementary Fig. S4.** Random effect plot for the wtN abiotic environment model, with wtN as the response and genus as the random effect.


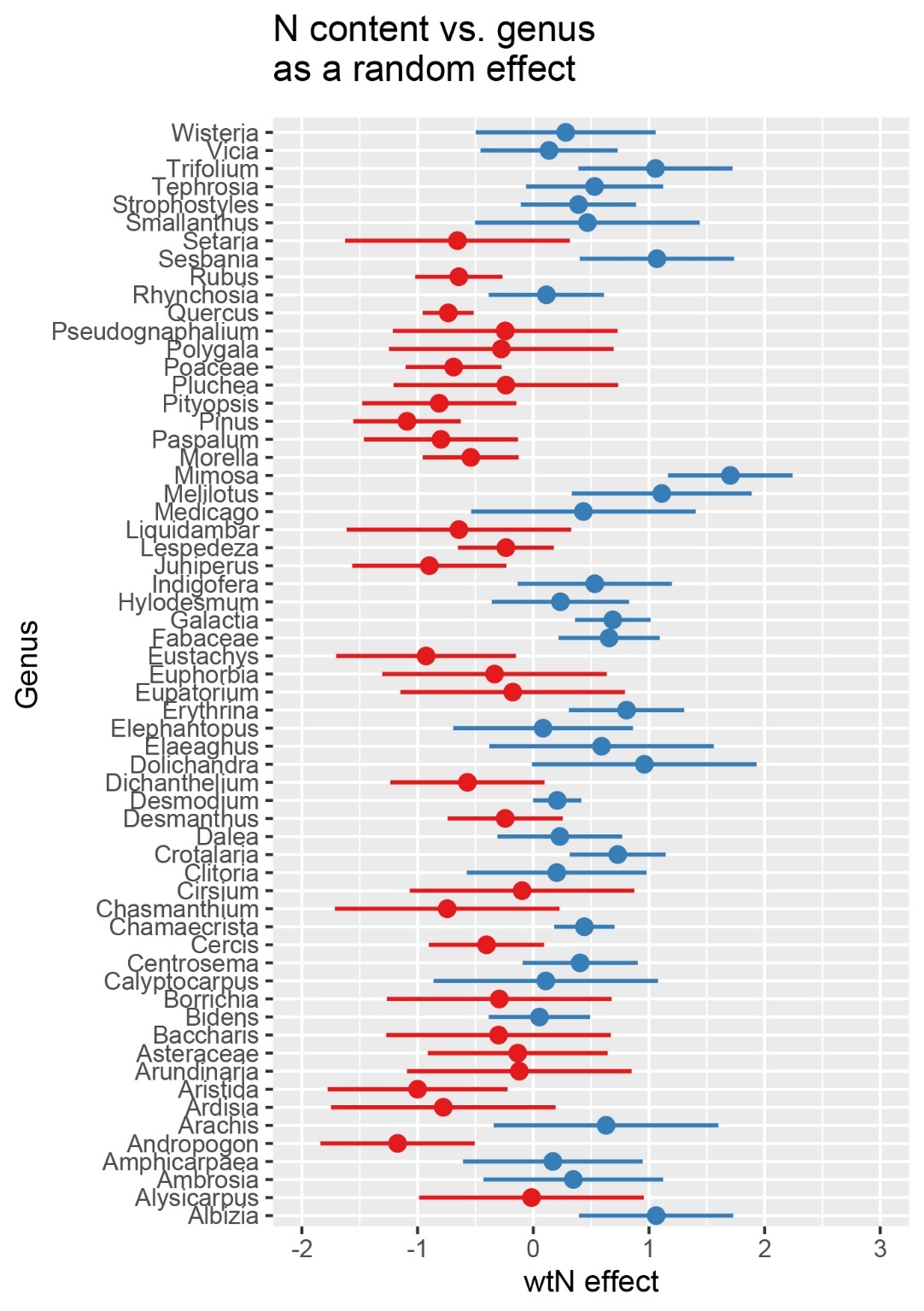


**Supplementary Fig. S5.** Random effect plot for the wtC abiotic environment model, with wtC as the response and genus as the random effect.


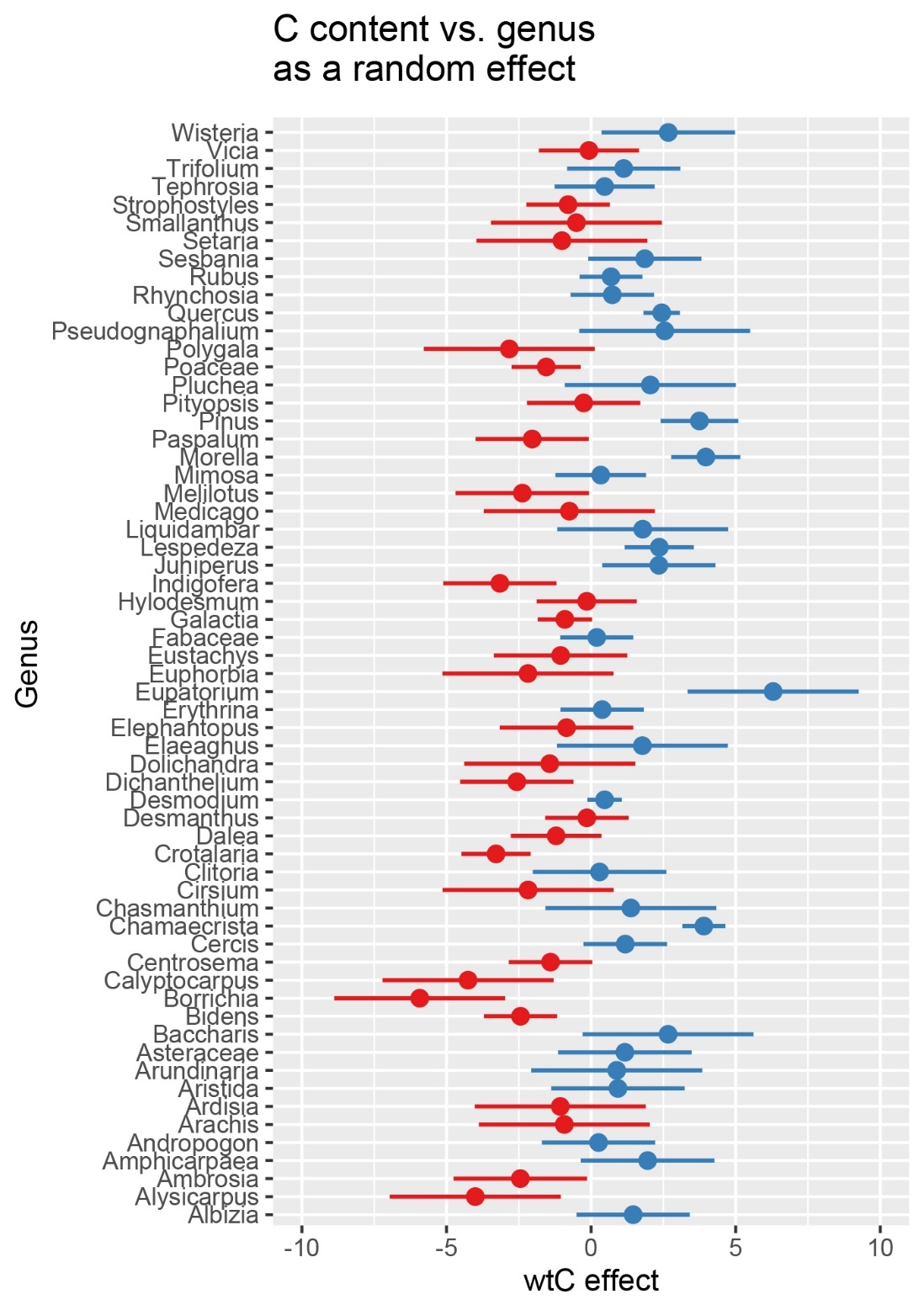


**Supplementary Fig. S5.** Random effect plot for the wtC abiotic environment model, with wtC  as the response and genus as the random effect.


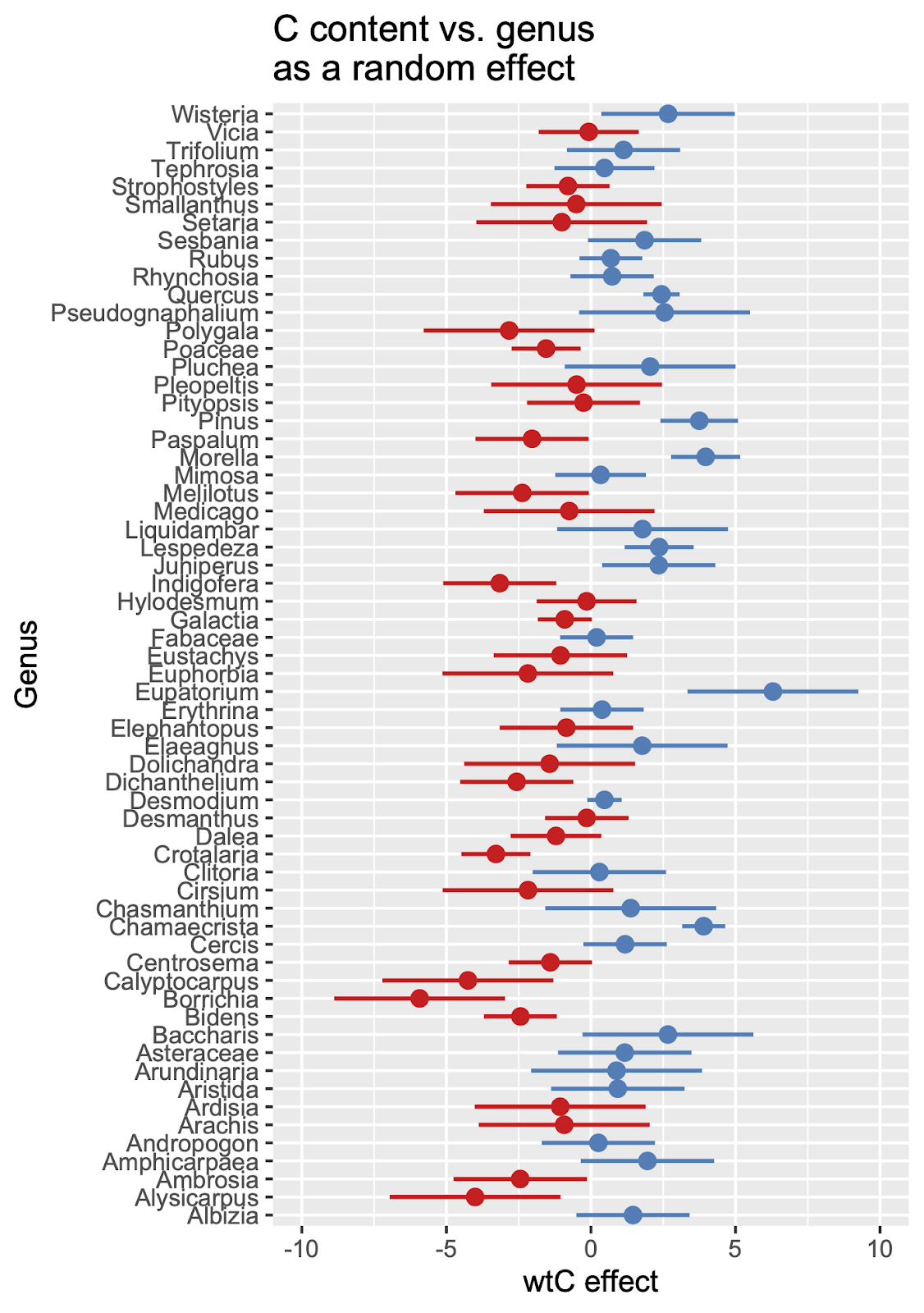


**Supplementary Fig. S6**


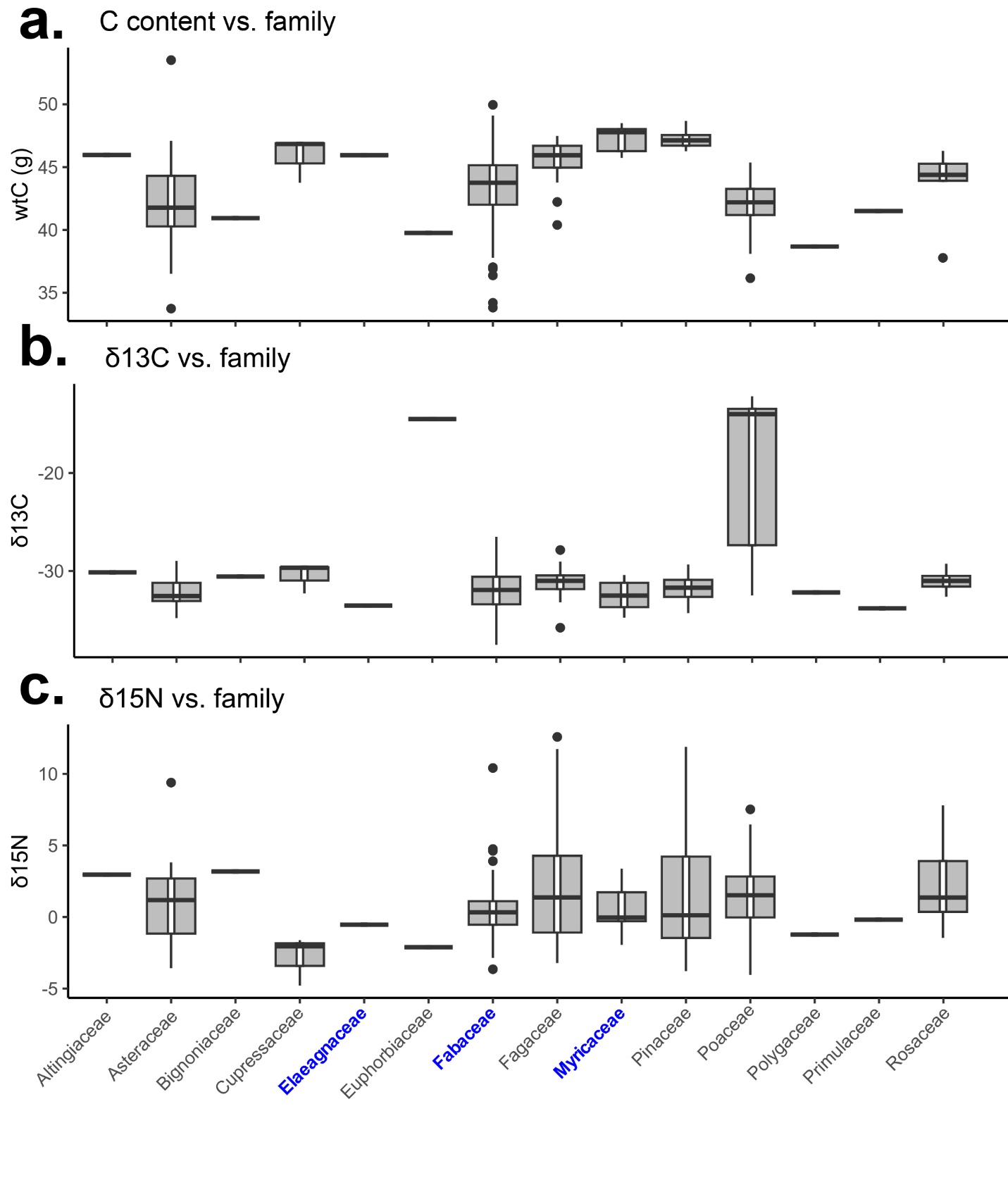


**Supplemental Table 1.** Sampled taxa and accession information.

| Family | Tribe | Species | mean d15N | range d15N | mean d13C | range d13C | mean wtN | range wtN | mean wtC | range wtC | Fixing Status | Native/  Exotic | # of sites present | Habitat type(s) | # of samples |
| --- | --- | --- | --- | --- | --- | --- | --- | --- | --- | --- | --- | --- | --- | --- | --- |
| Altingiaceae | N/A | *Liquidambar styraciflua* | 2.96 | 0 | -30.14 | 0 | 1.34 | 0 | 45.97 | 0 | 0 | N | 1 | Disturbed | 1 |
| Asteraceae | Heliantheae | *Ambrosia* sp. | 0.965 | 1.41 | -31.875 | 0.69 | 2.645 | 1.43 | 39.38 | 4.8 | 0 | N | 2 | Disturbed, Prairie | 2 |
| Asteraceae | ? | Asteraceae sp. | 2.47 | 1.48 | -33.625 | 0.75 | 1.98 | 0.22 | 44.79 | 0.96 | 0 | ? | 2 | Upland, Mixed | 2 |
| Asteraceae | Astereae | *Baccharis halimifolia* | 3.25 | 0 | -32.66 | 0 | 1.65 | 0 | 47.08 | 0 | 0 | N | 1 | Mixed | 1 |
| Asteraceae | Coreopsideae | *Bidens* sp. | 1.83 | 0 | -30.76 | 0 | 2.34 | 0 | 41.64 | 0 | 0 | N | 1 | Disturbed | 1 |
| Asteraceae | Coreopsideae | *Bidens alba* | -1.575 | 3.23 | -33.755 | 1.76 | 2.0825 | 1.58 | 39.2225 | 4.89 | 0 | N | 4 | Maritime, Saltmarsh, Mixed, Disturbed | 4 |
| Asteraceae | Coreopsideae | *Bidens pilosa* | -1.07 | 3.4 | -32.1767 | 1.55 | 2.983333 | 1.02 | 41.71667 | 3.17 | 0 | N | 3 | Disturbed | 3 |
| Asteraceae | Heliantheae | *Borrichia frutescens* | 9.39 | 0 | -29.62 | 0 | 2.1 | 0 | 33.74 | 0 | 0 | N | 1 | Maritime | 1 |
| Asteraceae | Cardueae | *Cirsium* sp. | 2.36 | 0 | -29.16 | 0 | 2.23 | 0 | 40.33 | 0 | 0 | ? | 1 | Swamp | 1 |
| Asteraceae | Astereae | *Pityopsis graminifolia* | 0.303333 | 3.91 | -32.5133 | 0.42 | 1.033333 | 0.61 | 43.17667 | 3.59 | 0 | N | 3 | Flatwoods, Upland | 3 |
| Asteraceae | Inuleae | *Pluchea* sp. | 3.77 | 0 | -32.03 | 0 | 1.46 | 0 | 46.31 | 0 | 0 | N | 1 | Flatwoods | 1 |
| Asteraceae | Gnaphalieae | *Pseudognaphalium obtusifolium* | 2.69 | 0 | -31.03 | 0 | 1.45 | 0 | 47.09 | 0 | 0 | N | 1 | Flatwoods | 1 |
| Asteraceae | Millerieae | *Smallanthus uvedalia* | -3.42 | 0 | -34.8 | 0 | 3.27 | 0 | 41.47 | 0 | 0 | N | 1 | CC Forest | 1 |
| Asteraceae | Veronieae | *Elephantopus elatus* | 0.985 | 1.35 | -29.35 | 0.72 | 2.265 | 1.17 | 42.58 | 0.8 | 0 | N | 2 | Upland | 2 |
| Asteraceae | Eupatorieae | *Eupatorium capillifolium* | 3.81 | 0 | -33.03 | 0 | 1.83 | 0 | 53.52 | 0 | 0 | N | 1 | Upland | 1 |
| Bignoniaceae | Bignonieae | *Dolichandra unguis-cati* | 3.18 | 0 | -30.57 | 0 | 4.2 | 0 | 40.94 | 0 | 0 | E | 1 | CC Forest | 1 |
| Cupressaceae | N/A | *Juniperus virginiana* | -2.82 | 3.15 | -30.5067 | 2.71 | 1.306667 | 1.28 | 45.85667 | 3.21 | 0 | N | 3 | Maritime, Saltmarsh, Disturbed | 3 |
| Elaeagnaceae | N/A | *Elaeagnus pungens* | -0.54 | 0 | -33.53 | 0 | 3.54 | 0 | 45.95 | 0 | 1 | E | 1 | Disturbed | 1 |
| Euphorbiaceae | Euphorbieae | *Euphorbia maculata* | -2.11 | 0 | -14.49 | 0 | 1.89 | 0 | 39.76 | 0 | 0 | N | 1 | Disturbed | 1 |
| Fabaceae | Ingeae | *Albizia julibrissin* | 1.346667 | 2 | -31.6267 | 5.54 | 3.713333 | 2.29 | 45.07 | 3.15 | 1 | E | 2 | Disturbed, Upland | 3 |
| Fabaceae | Desmodieae | *Alysicarpus vaginalis* | -1.69 | 0 | -33.94 | 0 | 2.46 | 0 | 36.91 | 0 | 1 | E | 1 | Disturbed | 1 |
| Fabaceae | Phaseoleae | *Amphicarpaea bracteata* | -0.68 | 0.54 | -32.6 | 0.44 | 2.67 | 0.94 | 44.77 | 1.78 | 1 | N | 1 | CC Forest | 2 |
| Fabaceae | Dalbergieae | *Arachis hypogaea* | -1.07 | 0 | -31.9 | 0 | 3.61 | 0 | 41.73 | 0 | 1 | E | 1 | Disturbed | 1 |
| Asteraceae | Heliantheae | *Calyptocarpus vialis* | -3.05 | 0 | -33.07 | 0 | 2.68 | 0 | 36.52 | 0 | 1 | E | 1 | Disturbed | 1 |
| Fabaceae | Phaseoleae | *Centrosema virginianum* | -1.08833 | 1.61 | -30.2317 | 4.96 | 2.68 | 1.53 | 41.4 | 3.03 | 1 | N | 4 | CC Forest, Maritime, Flatwoods | 6 |
| Fabaceae | Cercideae | *Cercis canadensis* | 4.275 | 8.76 | -32.5283 | 2.31 | 1.911667 | 2 | 44.07833 | 4.91 | 0 | N | 3 | CC Forest, Disturbed, Mixed | 6 |
| Fabaceae | Cassieae | *Chamaecrista fasciculata* | 0.536667 | 2.85 | -33.845 | 3.96 | 2.078333 | 1.5 | 45.27167 | 5.88 | 1 | N | 6 | Disturbed, Mixed, Prairie | 3 |
| Fabaceae | Cassieae | *Chamaecrista nictitans* | 0.451111 | 5.27 | -33.3956 | 5.56 | 2.836667 | 1.99 | 47.71833 | 4.81 | 1 | N | 9 | CC Forest, Mixed, Disturbed, Prairie, Upland | 18 |
| Fabaceae | Phaseoleae | *Clitoria mariana* | 2.32 | 1.34 | -28.01 | 0.34 | 2.43 | 0.14 | 44.045 | 0.37 | 1 | N | 1 | Upland | 2 |
| Fabaceae | Crotalarieae | *Crotalaria lanceolata* | -2.00667 | 2.49 | -31.78 | 5.05 | 3.6 | 3.9 | 43.78333 | 3.67 | 1 | E | 2 | Disturbed | 3 |
| Fabaceae | Crotalarieae | *Crotalaria pallida* | -1.46 | 0 | -34.45 | 0 | 5.51 | 0 | 43.69 | 0 | 1 | E | 1 | Disturbed | 1 |
| Fabaceae | Crotalarieae | *Crotalaria rotundifolia* | 1.953333 | 1.16 | -31.6 | 2.29 | 2.066667 | 1 | 36.57333 | 5.7 | 1 | N | 1 | Flatwoods | 2 |
| Fabaceae | Crotalarieae | *Crotalaria* sp. | 1.405 | 0.19 | -30.28 | 0.06 | 2.26 | 0.54 | 36.585 | 4.77 | 1 | N | 1 | Upland | 2 |
| Fabaceae | Amorpheae | *Dalea candida* | 1.535 | 0.29 | -28.97 | 1.3 | 1.94 | 0.42 | 39.13 | 2.66 | 1 | N | 1 | Prairie | 2 |
| Fabaceae | Amorpheae | *Dalea purpurea* | 0.26 | 0 | -26.51 | 0 | 2.6 | 0 | 40.55 | 0 | 1 | N | 1 | Prairie | 1 |
| Fabaceae | Amorpheae | *Dalea* sp. | 0.58 | 0.04 | -29.41 | 1.34 | 2.33 | 0.64 | 43.69 | 1.86 | 1 | ? | 1 | Upland | 2 |
| Fabaceae | Mimoseae | *Desmanthus illinoiensis* | 2.165 | 3.7 | -30.2617 | 4.29 | 1.996667 | 0.56 | 42.57833 | 2.82 | 1 | N | 3 | Disturbed, Prairie | 3 |
| Fabaceae | Desmodieae | *Desmodium ciliare* | 1.1 | 1.86 | -31.17 | 0.62 | 2.785 | 0.71 | 40.65 | 0.92 | 1 | N | 1 | Disturbed | 2 |
| Fabaceae | Desmodieae | *Desmodium floridanum* | 0.51125 | 2.02 | -32.4888 | 4.73 | 2.1675 | 1.4 | 43.4875 | 7.17 | 1 | N | 1 | Mixed | 2 |
| Fabaceae | Desmodieae | *Desmodium incanum* | -0.4125 | 2.98 | -33.195 | 1.43 | 2.4875 | 1.8 | 45.46 | 3.56 | 1 | E | 2 | Disturbed | 3 |
| Fabaceae | Desmodieae | *Desmodium rotundifolia* | 1.6825 | 2.28 | -32.9525 | 3.3 | 2.505 | 1.15 | 44.3675 | 1.49 | 1 | N | 1 | Mixed | 2 |
| Fabaceae | Desmodieae | *Desmodium* sp. | 0.6475 | 1.57 | -34.1975 | 3.34 | 2.11 | 0.37 | 42.9375 | 3.96 | 1 | ? | 3 | CC Forest, Mixed | 4 |
| Fabaceae | Desmodieae | *Desmodium strictum* | 0.621667 | 4.37 | -32.765 | 5.4 | 2.449167 | 2 | 43.315 | 3.55 | 1 | N | 3 | Disturbed, Flatwoods, Mixed | 6 |
| Fabaceae | Desmodieae | *Grona triflora (Desmodium triflorum)* | -1.0625 | 0.3 | -31.8675 | 1.86 | 3.3 | 2.89 | 44.3975 | 3.17 | 1 | E | 2 | Marititime, Disturbed | 4 |
| Fabaceae | Phaseoleae | *Erythrina herbacea* | 0.2 | 1.15 | -29.6533 | 7.03 | 3.331667 | 1.29 | 43.705 | 5.08 | 1 | N | 3 | Disturbed, Maritime, Upland | 6 |
| Fabaceae | Desmodieae | Fabaceae morph 1 | 0.2 | 1.3 | -34.04 | 0.4 | 3.77 | 1.36 | 44.7 | 2.24 | 1 | ? | 1 | Mixed | 2 |
| Fabaceae | Desmodieae | Fabaceae morph 2 | 1.7475 | 0.41 | -34.7275 | 1.48 | 2.245 | 1.41 | 43.9175 | 2.67 | 1 | ? | 2 | Mixed | 4 |
| Fabaceae | Diocleae | *Galactia elliottii* | -0.04 | 0 | -32.28 | 0 | 3.1 | 0 | 46.01 | 0 | 1 | N | 1 | Flatwoods | 1 |
| Fabaceae | Diocleae | *Galactia regularis* | -0.59125 | 1.87 | -30.93 | 3.77 | 3.0625 | 2.11 | 42.61625 | 4.4 | 1 | N | 1 | Disturbed | 2 |
| Fabaceae | Diocleae | *Galactia* sp. | -0.795 | 1.43 | -32.1725 | 2.69 | 3.6275 | 2.74 | 41.1875 | 2.34 | 1 | N | 2 | Marititime, Mixed | 4 |
| Fabaceae | Diocleae | *Galactia volubilis* | 1.66 | 2.46 | -31.425 | 1.35 | 1.795 | 0.29 | 42.015 | 0.45 | 1 | N | 1 | Disturbed | 2 |
| Fabaceae | Desmodieae | *Hylodesmum glutinosum* | 0.675 | 1.13 | -36.49 | 1.06 | 2.625 | 0.09 | 41.17 | 1.08 | 1 | N | 1 | CC Forest | 2 |
| Fabaceae | Desmodieae | *Hylodesmum nudiflorum* | 0.13 | 1.14 | -36.47 | 2.14 | 2.805 | 0.17 | 43.015 | 0.01 | 1 | N | 1 | CC Forest | 2 |
| Fabaceae | Indigofereae | *Indigofera hirsuta* | 0.49 | 0.72 | -32.06 | 0.4 | 1.91 | 0.04 | 38.8 | 0.18 | 1 | E | 1 | Mixed | 2 |
| Fabaceae | Indigofereae | *Indigofera spicata* | 0.1 | 0 | -30.67 | 0 | 5.05 | 0 | 40.16 | 0 | 1 | E | 1 | Disturbed | 1 |
| Fabaceae | Desmodieae | *Lespedeza cuneata* | -0.3125 | 0.8 | -32.4975 | 4.71 | 2.235 | 0.83 | 44.065 | 3.84 | 1 | E | 3 | CC Forest, Disturbed, Prairie | 4 |
| Fabaceae | Desmodieae | *Lespedeza sp.* | -0.004 | 2.85 | -30.5 | 1.87 | 1.736 | 1.2 | 46.842 | 3.48 | 1 | ? | 3 | Upland | 5 |
| Fabaceae | Trifolieae | *Medicago lupulina* | -0.24 | 0 | -31.87 | 0 | 3.26 | 0 | 42 | 0 | 1 | E | 1 | Disturbed | 1 |
| Fabaceae | Trifolieae | *Melilotus albus* | 0.245 | 0.27 | -29.31 | 1.06 | 3.385 | 1.81 | 38.81 | 3.54 | 1 | E | 1 | Prairie | 2 |
| Fabaceae | Mimoseae | *Mimosa pudica* | -0.01 | 0 | -31.07 | 0 | 3.04 | 0 | 45.12 | 0 | 1 | E | 1 | Disturbed | 1 |
| Fabaceae | Mimoseae | *Mimosa* sp. | 0.335 | 0.73 | -31.7825 | 1.7 | 4.4725 | 2.35 | 43.6475 | 6.73 | 1 | N | 2 | Sandhill | 4 |
| Fabaceae | Phaseoleae | *Rhynchosia* sp. | -0.11833 | 5.25 | -31.53 | 5.6 | 2.288333 | 1.05 | 44.22 | 1.81 | 1 | N | 3 | Upland, Mixed | 6 |
| Fabaceae | Sesbanieae | *Sesbania* sp. | -0.78333 | 1.56 | -30.99 | 0.62 | 3.516667 | 1.44 | 45.66 | 2.02 | 1 | N | 2 | Saltmarsh, Upland | 3 |
| Poaceae | Paniceae | *Setaria* sp. | 1.69 | 0 | -12.2 | 0 | 1.04 | 0 | 41.45 | 0 | 0 | ? | 1 | Saltmarsh | 1 |
| Fabaceae | Phaseoleae | *Strophostyles helvola* | -1.99 | 0.84 | -33.11 | 3.12 | 2.43 | 0.68 | 42.49 | 0.68 | 1 | N | 1 | Disturbed | 2 |
| Fabaceae | Phaseoleae | *Strophostyles* sp. | -1.355 | 0.93 | -29.31 | 4.01 | 3.315 | 1.2 | 42.0475 | 3.95 | 1 | N | 2 | Maritime | 4 |
| Fabaceae | Millettieae | *Tephrosia florida* | -1.25 | 0 | -32.88 | 0 | 3.5 | 0 | 42.57 | 0 | 1 | N | 1 | Flatwoods | 1 |
| Fabaceae | Millettieae | *Tephrosia* sp. | 0.64 | 0.6 | -28.8967 | 0.78 | 2.453333 | 0.45 | 44.56 | 1.18 | 1 | N | 2 | Upland | 3 |
| Fabaceae | Trifolieae | *Trifolium pratense* | 0.42 | 0.46 | -33.44 | 0.7 | 4.035 | 0.63 | 45.82 | 0.1 | 1 | E | 1 | Disturbed | 2 |
| Fabaceae | Trifolieae | *Trifolium repens* | -1.53 | 0 | -31.58 | 0 | 3.39 | 0 | 41.91 | 0 | 1 | E | 1 | Disturbed | 1 |
| Fabaceae | Fabeae | *Vicia acutifolia* | 0.87 | 0.66 | -30.64 | 1.54 | 2.545 | 0.43 | 41.385 | 1.25 | 1 | N | 1 | Saltmarsh | 1 |
| Fabaceae | Fabeae | *Vicia* sp. | 0.325 | 0.73 | -31.8 | 1.86 | 2.175 | 0.07 | 44.385 | 0.27 | 1 | ? | 1 | Mixed | 2 |
| Fabaceae | Millettieae | *Wisteria sinensis* | 0.635 | 0.77 | -28.9 | 1.68 | 2.23 | 0.5 | 45.28 | 2 | 1 | E | 1 | Prairie | 2 |
| Fagaceae | N/A | *Quercus falcata* | 6.19 | 3.82 | -31.185 | 1.33 | 1.565 | 0.37 | 46.99 | 0.38 | 0 | N | 2 | Upland | 2 |
| Fagaceae | N/A | *Quercus hemispherica* | 0.1825 | 8.34 | -30.95 | 0.85 | 1.715 | 0.18 | 47.23 | 0.39 | 0 | N | 2 | Disturbed, Mixed | 2 |
| Fagaceae | N/A | *Quercus laevis* | -1.9 | 0 | -29.85 | 0 | 0.9 | 0 | 45.95 | 0 | 0 | N | 1 | Upland | 1 |
| Fagaceae | N/A | *Quercus margaretta* | 1.38 | 0 | -30.71 | 0 | 1.57 | 0 | 46.7 | 0 | 0 | N | 1 | Upland | 1 |
| Fagaceae | N/A | *Quercus minima* | 1.57 | 0 | -31.41 | 0 | 1.38 | 0 | 44.62 | 0 | 0 | N | 1 | Upland | 1 |
| Fagaceae | N/A | *Quercus nigra* | -1.39 | 0 | -32.52 | 0 | 1.97 | 0 | 46.64 | 0 | 0 | N | 1 | Disturbed | 1 |
| Fagaceae | N/A | *Quercus* sp. | 3.213333 | 15.79 | -31.3986 | 7.93 | 1.563333 | 0.76 | 45.10381 | 6.93 | 0 | N | 21 | CC Forest | 21 |
| Fagaceae | N/A | *Quercus virginiana* | -1.05667 | 1.12 | -30.5733 | 0.94 | 1.36 | 0.64 | 46.49 | 1.33 | 0 | N | 3 | Mix, Maritime, Upland | 3 |
| Fagaceae | N/A | *Rubus cuneifolius* | 1.581111 | 7.75 | -30.9144 | 3.35 | 1.433333 | 0.86 | 44.10778 | 8.51 | 0 | N | 9 | Flatwoods, Upland, Swamp | 9 |
| Myricaceae | N/A | *Morella cerifera* | 0.03 | 0.14 | -32.055 | 3.27 | 1.495 | 0.33 | 47.905 | 0.25 | 1 | N | 2 | Disturbed, Mixed | 2 |
| Myricaceae | N/A | *Morella pumila* | -0.3 | 0 | -31.21 | 0 | 1.33 | 0 | 48.49 | 0 | 1 | N | 1 | Flatwoods | 1 |
| Myricaceae | N/A | *Morella* sp. | 0.69 | 5.31 | -32.7633 | 3.57 | 1.758333 | 0.37 | 46.955 | 2.51 | 1 | N | 5 | Flatwoods, Maritime, Mixed, Upland | 10 |
| Pinaceae | N/A | *Pinus elliotii* | 11.88 | 0 | -31.39 | 0 | 0.81 | 0 | 48.68 | 0 | 0 | N | 1 | Flatwoods | 1 |
| Pinaceae | N/A | *Pinus palustris* | 0.12 | 2.03 | -31.71 | 1.29 | 0.69 | 0.32 | 46.98 | 0.3 | 0 | N | 2 | Mixed, Upland | 2 |
| Pinaceae | N/A | *Pinus* sp. | 1.99 | 4.84 | -31.82 | 4.94 | 1.185 | 0.01 | 46.96 | 0.74 | 0 | N | 2 | CC Forest, Swamp | 2 |
| Pinaceae | N/A | *Pinus taeda* | 0.13 | 7.8 | -32.64 | 0.08 | 1.165 | 0.43 | 47.03 | 1.52 | 0 | N | 2 | Mixed, Upland | 2 |
| Poaceae | Andropogoneae | *Andropogon* sp. | 5.24 | 0 | -13.99 | 0 | 0.95 | 0 | 43.06 | 0 | 0 | N | 1 | Swamp | 1 |
| Poaceae | Andropogoneae | *Andropogon glomeratus* | 2.31 | 1.04 | -13.68 | 0.46 | 0.76 | 0.22 | 43.855 | 1.17 | 0 | N | 2 | Maritime, Flatwoods | 2 |
| Primulaceae | N/A | *Ardisia crenata* | -0.19 | 0 | -33.81 | 0 | 1.1 | 0 | 41.51 | 0 | 0 | E | 1 | Disturbed | 1 |
| Poaceae | Aristideae | *Aristida beyrichiana* | -0.74 | 2 | -13.615 | 1.03 | 0.755 | 0.31 | 44.86 | 1 | 0 | N | 2 | Upland | 2 |
| Poaceae | Arundinarieae | *Arundinaria gigantea* | 1.5 | 0 | -27.37 | 0 | 1.97 | 0 | 44.3 | 0 | 0 | N | 1 | Mixed | 1 |
| Poaceae | Chasmanthieae | *Chasmanthium sessiliflorum* | 1.52 | 0 | -29.97 | 0 | 1.16 | 0 | 45.33 | 0 | 0 | N | 1 | Disturbed | 1 |
| Poaceae | Paniceae | *Dichanthelium* sp. | 2.056667 | 2.4 | -30.9867 | 3.42 | 1.543333 | 0.64 | 40.45333 | 2.13 | 0 | ? | 2 | Disturbed, Upland | 2 |
| Poaceae | Cynodonteae | *Eustachys* sp. | -2.46 | 3.16 | -14.61 | 0.3 | 1.115 | 0.31 | 41.61 | 0.84 | 0 | N | 2 | Maritime, Disturbed | 2 |
| Poaceae | Paspaleae | *Paspalum notatum* | 3.145 | 6.35 | -13.055 | 0.29 | 1.215 | 0.41 | 41.835 | 0.73 | 0 | E | 1 | Mixed | 1 |
| Poaceae | Paspaleae | *Paspalum* sp. | 7.52 | 0 | -14.06 | 0 | 1.37 | 0 | 38.83 | 0 | 0 | ? | 1 | Disturbed | 1 |
| Poaceae | ? | *Poaceae sp.* | 1.305556 | 9.14 | -17.3056 | 19.61 | 1.477778 | 1.23 | 41.37778 | 7.36 | 0 | ? | 9 | Saltmarsh, CC Forest, Mixed, Swamp, Disturbed, Prairie, Upland | 9 |
| Polygalaceae | Polygaleae | *Polygala lutea* | -1.23 | 0 | -32.19 | 0 | 1.39 | 0 | 38.68 | 0 | 0 | N | 1 | Flatwoods | 1 |
| Rosaceae | Rubeae | *Rubus* sp. | 5.91 | 3.76 | -31.82 | 1.16 | 1.64 | 0.4 | 44.375 | 1.05 | 0 | N | 2 | Disturbed | 2 |
